# The Synergistic Effects of 5-Aminosalicylic Acid and Vorinostat in the Treatment of Ulcerative Colitis

**DOI:** 10.3389/fphar.2021.625543

**Published:** 2021-05-21

**Authors:** Long He, Shuting Wen, Zhuotai Zhong, Senhui Weng, Qilong Jiang, Hong Mi, Fengbin Liu

**Affiliations:** ^1^ The First Clinical College, Guangzhou University of Chinese Medicine, Guangzhou, China; ^2^ Lingnan Medical Reserch Center of Guangzhou University of Chinese Medicine, Guangzhou, China; ^3^ Department of Gastroenterology, The First Affiliated Hospital of Guangzhou University of Chinese Medicine, Guangzhou, China; ^4^ Baiyun Hospital of the First Affiliated Hospital of Guangzhou University of Chinese Medicine, Guangzhou, China

**Keywords:** ulcerative colitis, 5-ASA, SAHA, protein-metabolite interactions, butyric acid, synergistic effects

## Abstract

**Background:** The drug 5*-*aminosalicylic acid (5-ASA) is the first-line therapy for the treatment of patients with mild-to-moderate ulcerative colitis (UC). However, in some cases, 5-ASA cannot achieve the desired therapeutic effects. Therefore, patients have to undergo therapies that include corticosteroids, monoclonal antibodies or immunosuppressants, which are expensive and may be accompanied by significant side effects. Synergistic drug combinations can achieve greater therapeutic effects than individual drugs while contributing to combating drug resistance and lessening toxic side effects. Thus, in this study, we sought to identify synergistic drugs that can act synergistically with 5-ASA.

**Methods:** We started our study with protein-metabolite analysis based on peroxisome proliferator-activated receptor gamma (PPARG), the therapeutic target of 5-ASA, to identify more additional potential drug targets**.** Then, we further evaluated the possibility of their synergy with PPARG by integrating Kyoto Encyclopedia of Genes and Genome (KEGG) pathway enrichment analysis, pathway-pathway interaction analysis, and semantic similarity analysis. Finally, we validated the synergistic effects with *in vitro* and *in vivo* experiments.

**Results:** The combination of 5-ASA and vorinostat (SAHA) showed lower toxicity and mRNA expression of p65 in human colonic epithelial cell lines (Caco-2 and HCT-116), and more efficiently alleviated the symptoms of dextran sulfate sodium (DSS)-induced colitis than treatment with 5-ASA and SAHA alone.

**Conclusion:** SAHA can exert effective synergistic effects with 5-ASA in the treatment of UC. One possible mechanism of synergism may be synergistic inhibition of the nuclear factor kappa B (NF-kB) signaling pathway. Moreover, the metabolite-butyric acid may be involved.

## Introduction

Ulcerative colitis (UC) is a chronic inflammatory disease affecting the colon, with increasing prevalence in recent years (Ungaro et al., 2017). Treatments for UC mainly include 5-aminosalicylic acid (5-ASA) drugs, biological drugs, corticosteroids, and immunosuppressants (Doyle and King, 1989). Among these, 5-ASA drugs are the main choice for the treatment of patients with mild-to-moderate UC (Pedersen and Brynskov, 2010), mainly because of their efficacy and safety. However, a subset of patients may not respond to 5-ASA, therefore, they can only be treated with corticosteroids or biological drugs (Bressler et al., 2015). Patients with UC usually require long-term medication to maintain efficacy, but long-term treatment with corticosteroids may produce acquired drug resistance and significant side effects (Bressler et al., 2015). Biological drugs, such as anti-tumor necrosis factor (TNF)-α therapies, are very expensive. Thus, it is necessary to develop a viable solution to enhance efficacy and reduce drug resistance to 5-ASA.

To treat certain complex diseases, such as cancer, cardiovascular diseases and type II diabetes, drug combinations have been developed (Kreek and Balint, 1980). Enhanced synergistic and antagonistic effects can be created by different types of drug combinations (Jia et al., 2009), and can contribute to combating drug resistance and disease heterogeneity (Hu et al., 2019). Among these, synergistic drug combinations present a prosperous future (Sheng et al., 2018) as they can achieve greater therapeutic effects than the drugs individually (Chou, 2006), while reducing side effects and drug resistance at the same time (Nowak-Sliwinska et al., 2016). Therefore, we hypothesized that identifying synergistic drugs for 5-ASA may be an effective solution to solve the problem of its insufficient efficacy and drug resistance in certain cases.

Based on previous studies, the crucial step for identifying synergistic drugs may be the discovery of potential drug candidates (Sun et al., 2015). In recent years, protein-protein interactions (PPIs) have been considered promising targets in drug discovery (Murakami et al., 2017). This is mainly due to the important roles of proteins in the regulation of biological systems and disease development (Scott et al., 2016). However, proteins can also be associated indirectly with downstream reactions through metabolites (Casas et al., 2019). In addition, recent studies have shown that many metabolites can function intracellularly or act directly as signaling molecules (Husted et al., 2017). For instance, the β-hydroxybutyrate (BHB) ketone can provide pivotal energy when the body's glucose supply is too low (Newman and Verdin, 2017). Furthermore, metabolites can also regulate protein function by acting as protein modifiers (Li et al., 2013). In fact, many signaling events are governed by intermediate metabolites rather than PPIs (Li et al., 2013; Casas et al., 2019). In addition, several studies have reported that metabolites play a vital role in drug discovery (Bertrand et al., 2014; Wishart, 2016). Subsequently, protein-metabolite interactions have emerged as new leads for drug discovery (Casas et al., 2019; Mateus et al., 2020).

In this manuscript, we sought to discover synergistic drugs for 5-ASA to solve the problem of drug resistance and insufficient efficacy that appear in some cases. To identify potential drug candidates, we constructed a network of protein-metabolite interactions and filtered out undruggable targets. Then, we integrated KEGG pathway enrichment analysis, pathway-pathway interaction analysis, and semantic similarity analysis to screen for the most effective targets (Supplementary Figure S1). Through the Therapeutic Target database (TTD), we finally identified that SAHA, a histone deacetylase (HDAC) inhibitor (Eckschlager et al., 2017), can work synergistically with 5-ASA. Moreover, we validated their synergistic effects by experiments by *in vitro* and *in vivo* experiments. Notably, PPARG, a key factor in mucosal homeostasis, is the therapeutic target of 5-ASA (Bouguen et al., 2015). Thus, we started our study with protein-metabolite analysis based on PPARG.

## Results

### Protein-Metabolite Analysis and Network Construction

**FIGURE 1:**
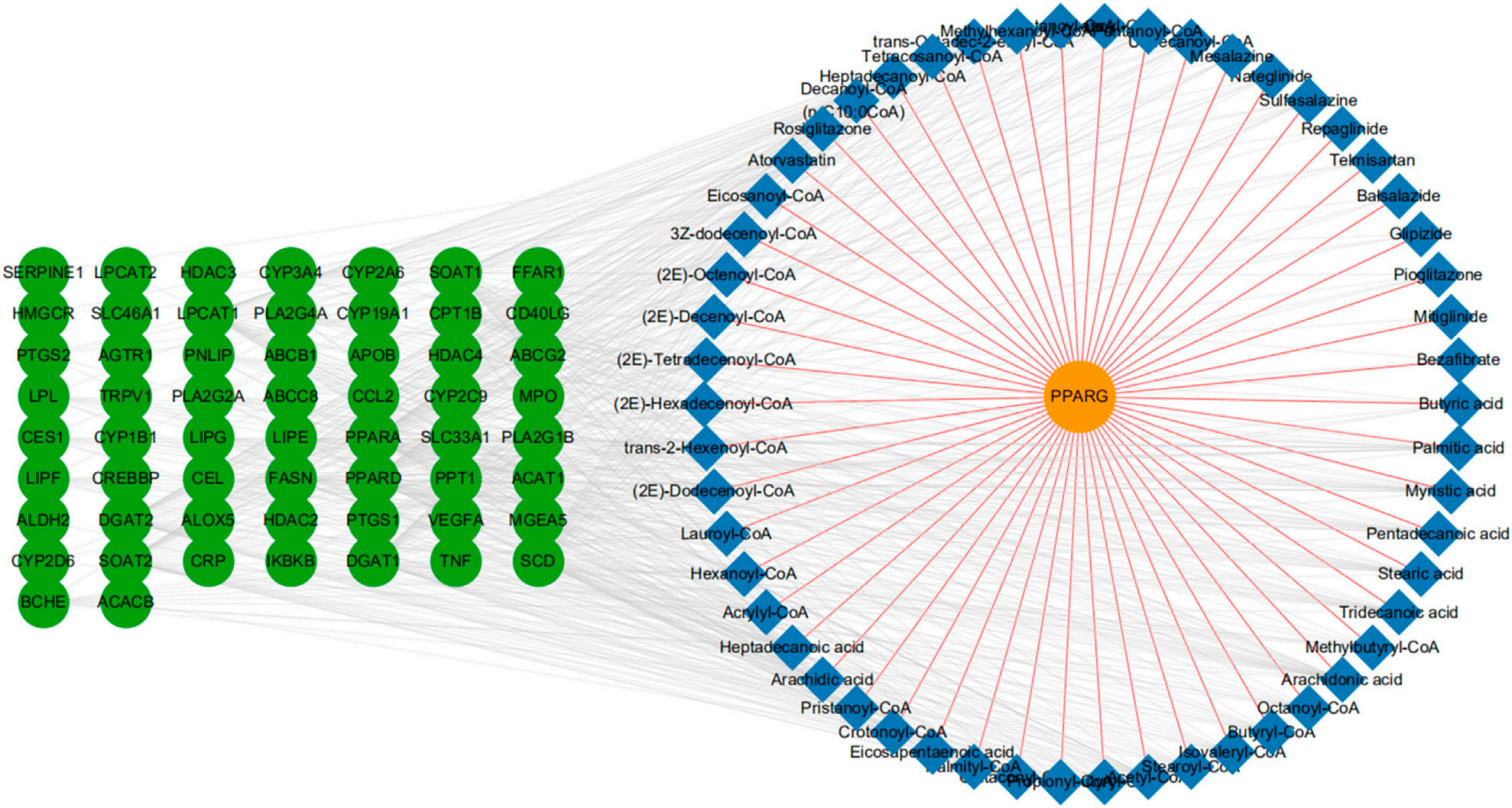
Protein-metabolite network construction. A full network based on the primary protein, PPARG (orange node), associated metabolites (blue node), and related druggable proteins (green node). We also show PPARG-metabolite interactions (brown-yellow edges), and metabolite-protein interactions (light gray edges).

To identify additional potential drug targets that work synergistically on PPARG, we conducted protein-metabolite interaction analysis through the Human Metabolome Database (HMDB). The HMDB is a database that can provide information about metabolites and their associated proteins (Wishart et al., 2018). In total, with 54 metabolite entries, we established 359 proteins interacting indirectly with PPARG. Next, we further limited the range of potential targets by filtering undruggable target proteins through the TTD (Yang et al., 2016). Subsequently, 63 druggable target proteins (those currently in clinical trials or used in the clinic) were selected (Supplementary Table S1). Finally, we integrated the protein-metabolite interactions and built a two-layered network showing the interactions among the associated proteins, metabolite entries, and our seed target-PPARG (Figure 1).

### KEGG Pathway Enrichment Analysis and Pathway-Pathway Interaction Analysis

**FIGURE 2:**
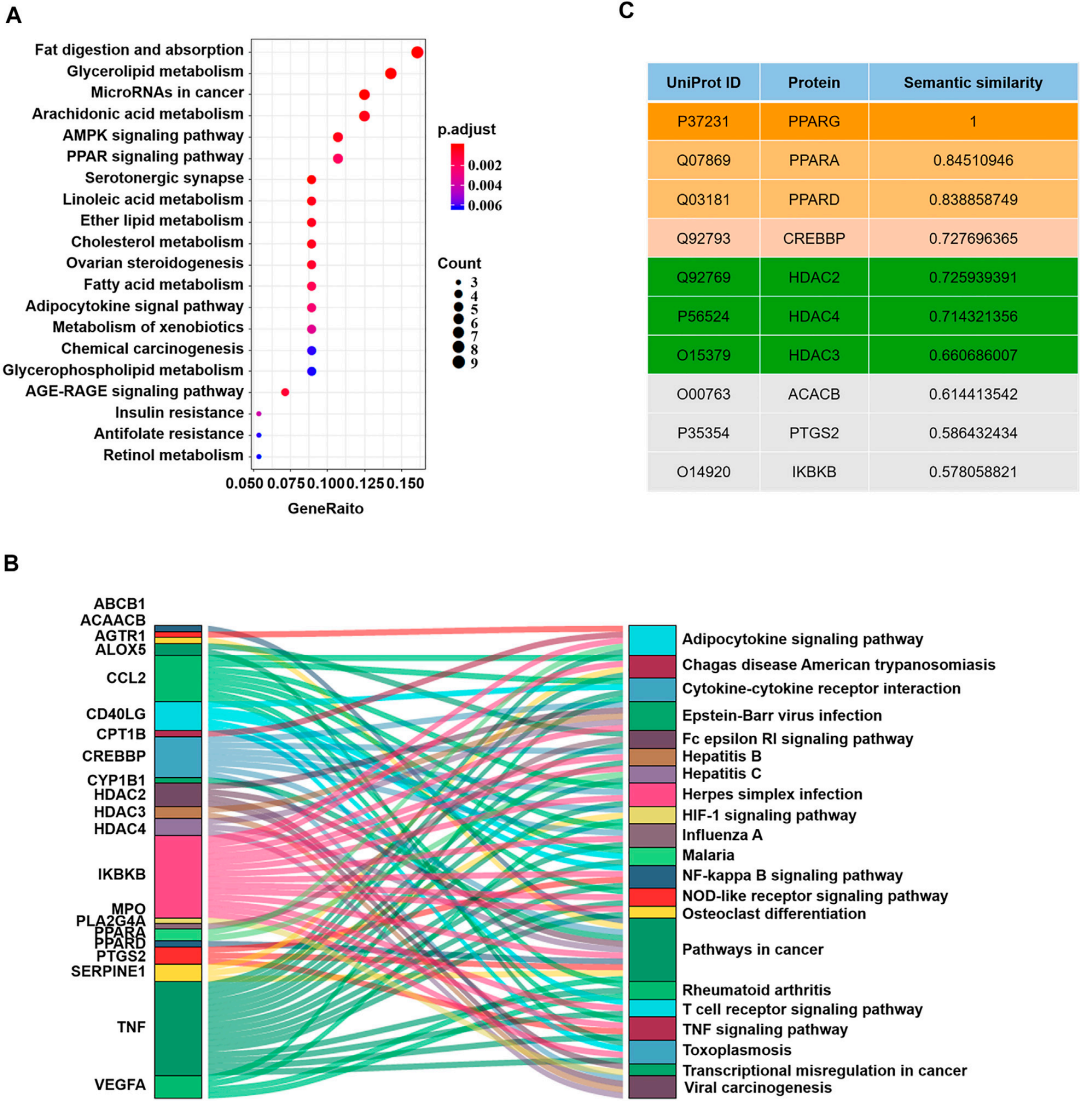
Measurement of the possibility of synergy. **(A)** KEGG pathway enrichment analysis on druggable target proteins. **(B)** Sankey plots of the pathways enriched for the druggable genes. **(C)** The top 10 candidate target proteins with semantic similarity scores and their UniProt IDs.

Given the potential drug targets, we next evaluated the possibility of synergy between these targets and PPARG. It has been reported that different drug targets involved in the same or associated pathways may produce synergistic effects (Jia et al., 2009). Since PPARG participates in the regulation of UC primarily by inhibiting the nuclear factor-kB (NF-kB) signaling pathway (Cao et al., 2018), drug targets involved in the NF-kB pathway or related pathways can function synergistically with PPARG. Moreover, Chen et al. proposed that pathways can interact with each other by gene-overlapping, functional-related and protein-protein interactions, and gene-overlapping pathway-pathway interactions suggesting more potential for predicting drug synergy (Chen D. et al., 2016). Therefore, we conducted KEGG pathway enrichment analysis on these 63 protein targets (Figure 2A). Consequently, pathways such as “Pathways in cancer”, “Viral carcinogenesis”, “Epstein-Barr virus infection”, and “Adipocytokine signaling pathway” were identified to interact with the NF-κB pathway *via* gene-overlapping (Figure 2B). Collectively, 20 targets involved in these pathways can function synergistically with PPARG (Figure 2B).

### Semantic Similarity of Gene Ontology Terms Analysis

Gene Ontology (GO) is a commonly used bioinformatics resource that provides information on the study of protein-protein interactions (Jain and Bader, 2010; Gene Ontology, 2015). Two proteins with similar functions could be further estimated by the geometric mean of semantic similarities in molecular function (MF) and component (CC) of each GO term pair (Wang et al., 2011; Han et al., 2013). Since functional similarity reflects the strength of the relationship between two proteins (Han et al., 2013), by evaluating the functional similarities between each potential target protein and PPARG through the semantic GOSemSim package using the Wang process (Yu et al., 2010), we further narrowed the range of candidate target protein. Finally, we obtained a rank list of functional similarity scores between the candidate target proteins and PPARG (Figure 2C; Supplementary Table S2).

Peroxisome proliferator-activated receptor alpha (PPARA), peroxisome proliferator-activated receptor delta (PPARD), histone deacetylase 2 (HDAC2), CREB binding protein (CREBBP), histone deacetylase 4 (HDAC4) and histone deacetylase 3 (HDAC3) received the highest scores, suggesting that they were the most likely to synergize with PPARG. However, PPARA is more likely to interfere with the NF-kB pathway in primary smooth muscle cells and hepatocytes rather than intestinal epithelial cells (Bougarne et al., 2018); PPARD is more involved in lipid metabolism; and histone deacetylases (HDACs) are upstream of CREBBP and can inhibit CREBPP (Jia et al., 2018). Thus, HDACs could be ideal candidate target proteins to work synergistically with PPARG in UC. Therefore, we next validated our predictions through experiments.

### Validation of the Synergistic Effects of SAHA and 5-ASA in Human Colonic Epithelial Cell Lines

**FIGURE 3:**
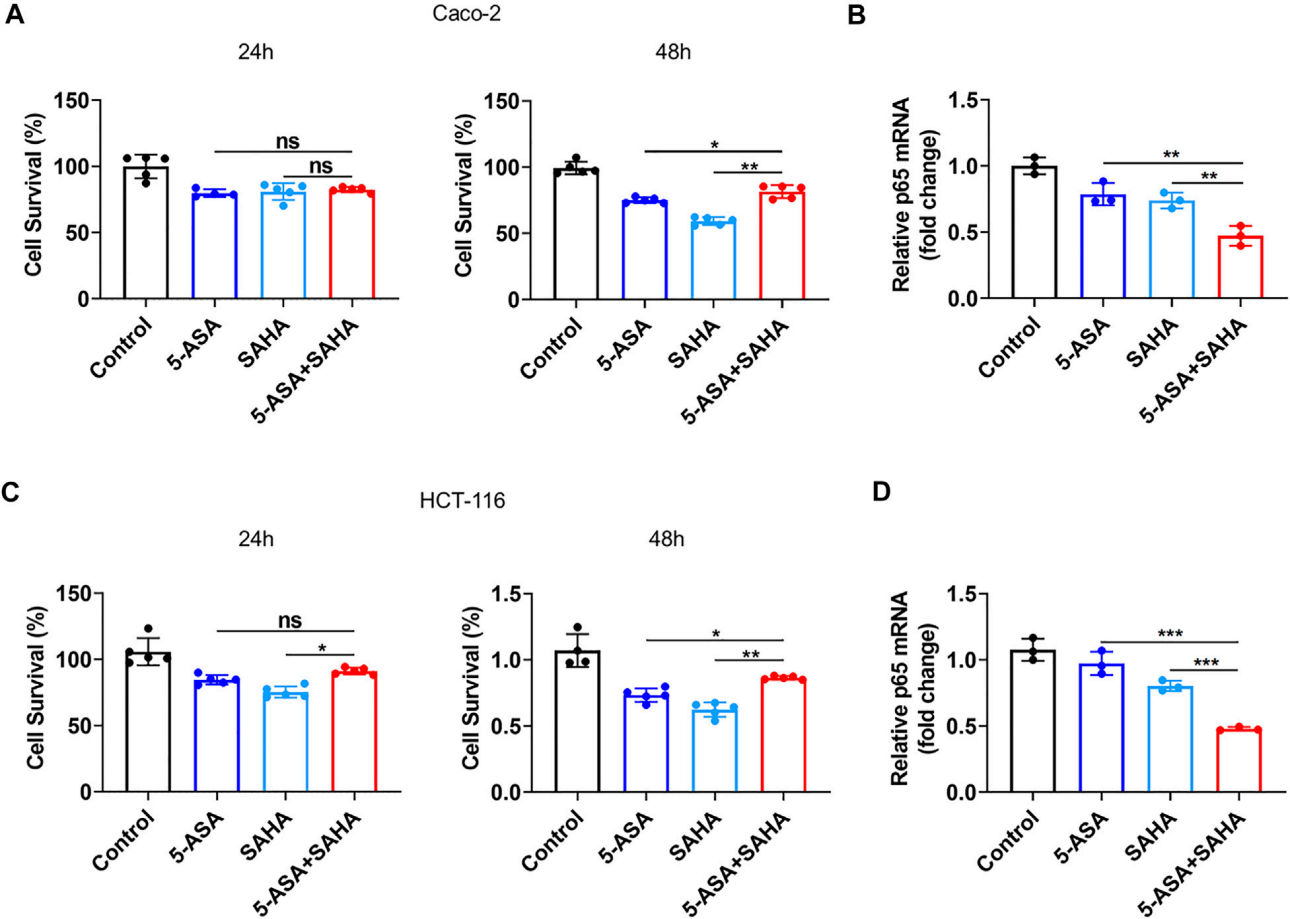
Validation of the prediction results *in vivo*. **(A,C)** CCK eight analysis was performed to evaluate the toxicity of 5-ASA (30 mM) and SAHA (5 µM) alone or in combination in Caco-2 cells or HCT-116 cells after 24 and 48 h of treatment; **(B,D)** The p65 mRNA levels were examined in Caco-2 cells and HCT-116 cells treated with 5-ASA and SAHA in . The data shown are the mean ± SD and represent three independent experiments, *p* values were calculated using an unpaired *t*-test, *p < 0.05, **p < 0.01, ***p < 0.001, ns = not significant.

We selected human colon adenocarcinoma cells (Caco-2) and colon cancer cells (HCT-116) to validate the synergistic effects of SAHA and 5-ASA. First, the cytotoxic effects of the PPARG agonist 5-ASA (30 mM) and HDACs inhibitor SAHA (5 µM) on Caco-2 cells were observed after 24 and 48 h of treatment. Notably, the combination of 5-ASA and SAHA from the CCK-8 assay had a lower cytotoxic effect than that of Caco-2 cells treated with 5-ASA or SAHA alone after 48 h of treatment (Figure 3A). In addition, when combining 5-ASA with SAHA, the mRNA expression of NF-kB (p65) was significantly reduced compared with that when the cells were individually treated with 5-ASA or SAHA (Figure 3B). Similarly, treatment with both 5-ASA and SAHA showed lower cytotoxic effects (Figure 3C) and p65 mRNA expression (Figure 3D) than individual treatment with 5-ASA or SAHA alone in HCT-116 cells. These findings displayed that the combined use of 5-ASA and SAHA can reduce drug cytotoxicity and synergistically inhibit the NF-kB signaling pathway.

### Synergistic Effects of SAHA and 5-ASA in Experimental Colitis

**FIGURE 4:**
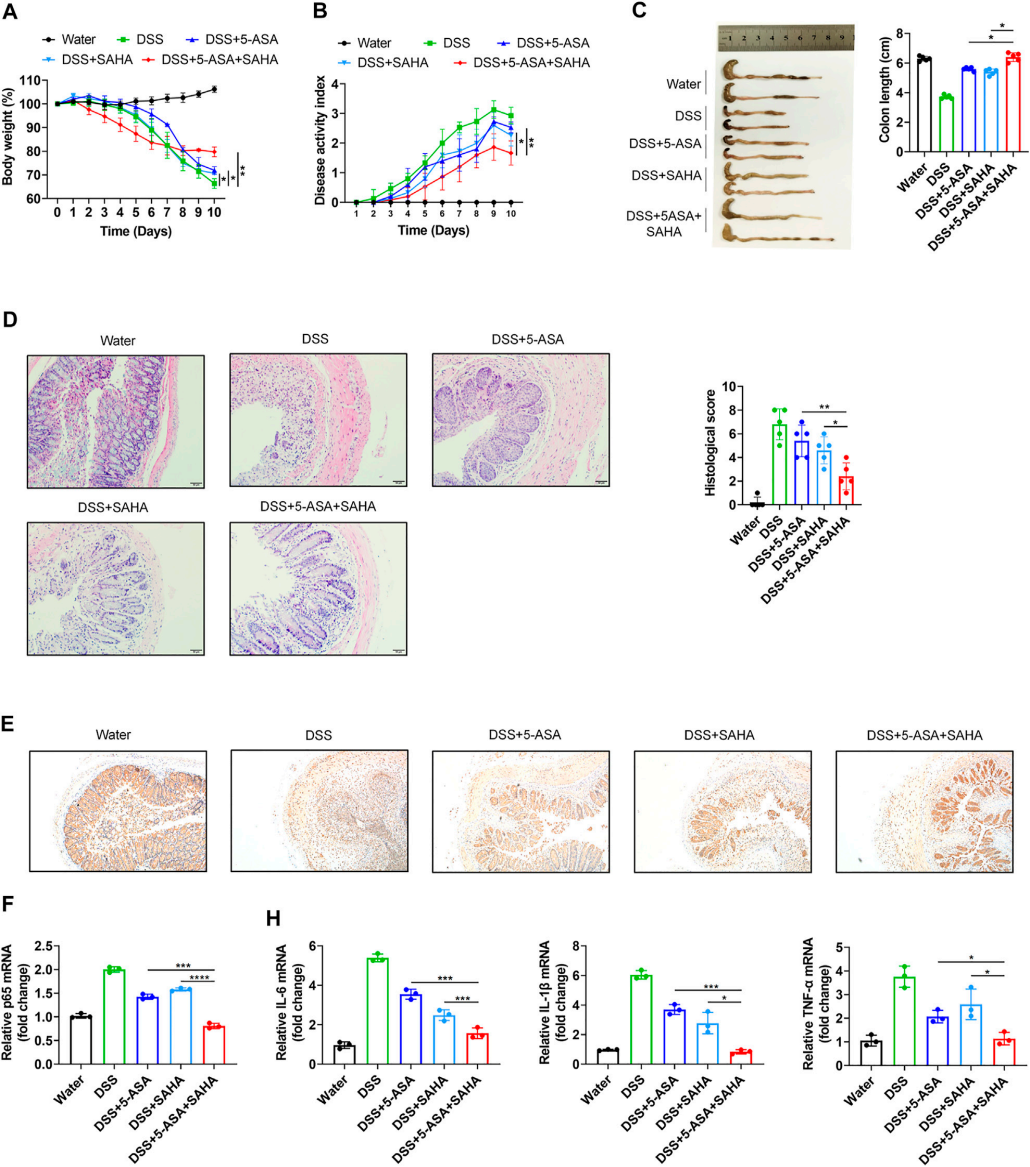
*In vitro* validation of the synergetic effects of 5-ASA and SAHA on DSS-induced colitis mice. B57BL/6 mice were administered 2.5% DSS in drinking water for 7 days, followed by 3 days of water (n = 5 per group). **(A)** Body weight, **(B)** DAI score, **(C)** colon length, **(D)** HE staining (200 × magnification) and histological scores of colitis mice treated with 5-ASA (100 mg/kg/day) and SAHA (200 mg/kg/day) in combination or individually. **(E)** Immunohistochemistry of p65 and **(F)** the p65, **(G)** IL-6, IL-1β, and TNF-α mRNA levels in colonic tissues. The data shown are the mean ± SD and represent three independent experiments, *p* values were calculated using unpaired *t*-test, *p < 0.05, **p < 0.01, ***p < 0.001, ns = not significant.

We next further confirmed the synergistic effects of 5-ASA and SAHA in experimental colitis. DSS-induced colitis mice were orally treated with either 5-ASA (100 mg/kg/day) or SAHA (200 mg/kg/day), and 5-ASA and SAHA combined treatment. Treatment with 5-ASA or SAHA reduced DSS-induced colitis symptoms, including weight loss. (Figure 4A), disease activity index (DAI) (Figure 4B), colon length (Figure 4C) and histological score (Figure 4D). While combination therapy was more effective in relieving these symptoms, our findings revealed that the combined use of 5-ASA and SAHA in the treatment of experimental colitis had a synergistic impact. We then sought to determine whether 5-ASA and SAHA play a synergistic role in experimental colitis because of their synergistic regulation of the NF-kB signaling pathway. Through immunohistochemical analysis, we detected p65 expression in colon tissues and found that the combined use of 5-ASA and SAHA significantly reduced p65 expression compared with 5-ASA or SAHA (Figure 4E). In addition, combined treatment showed lower mRNA expression of p65 compared with the single treatment (Figure 4F). The expression levels of downstream inflammatory factors such as IL-6, IL-1β, and TNF-α of the NF-kB signaling pathway were also significantly reduced (Figure 4G). Collectively, our findings indicate that SAHA and 5-ASA can exert a synergistic effect in the treatment of experimental colitis by synergistically inhibiting the NF-kB signaling pathway.

### Synergistic Effects of SAHA and 5-ASA in the Treatment of Experimental Colitis May be Associated With Butyric Acid

**FIGURE 5:**
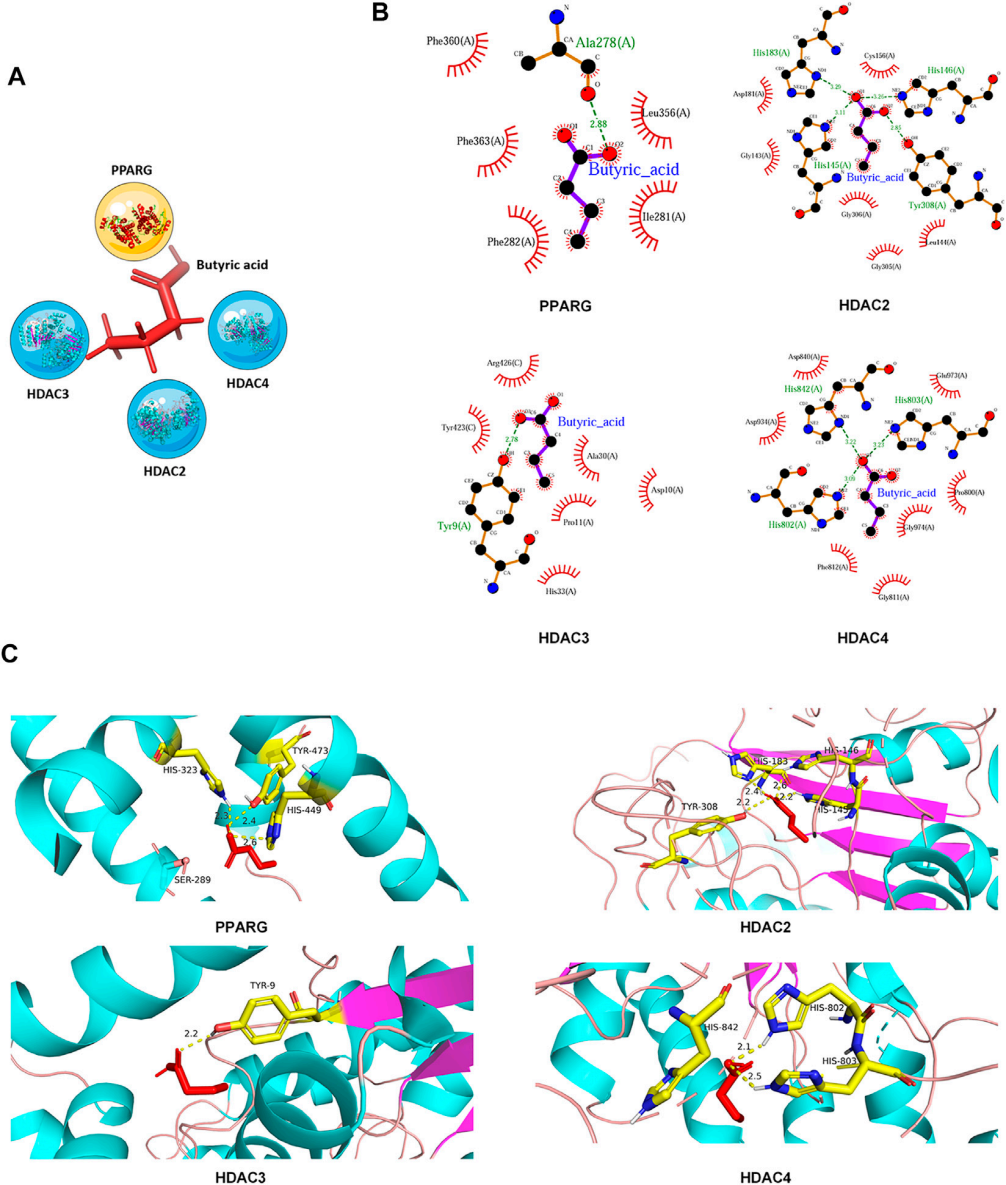
PPARG and HDACs can interact with butyric acid. **(A)** Network of interaction among PPARG, HDACs and the metabolite-butyric acid. **(B)** 2D and **(C)** 3D versions of the molecular docking of butyric acid to PPARG, HDAC2, HDAC3, and HDAC4.

**FIGURE 6:**
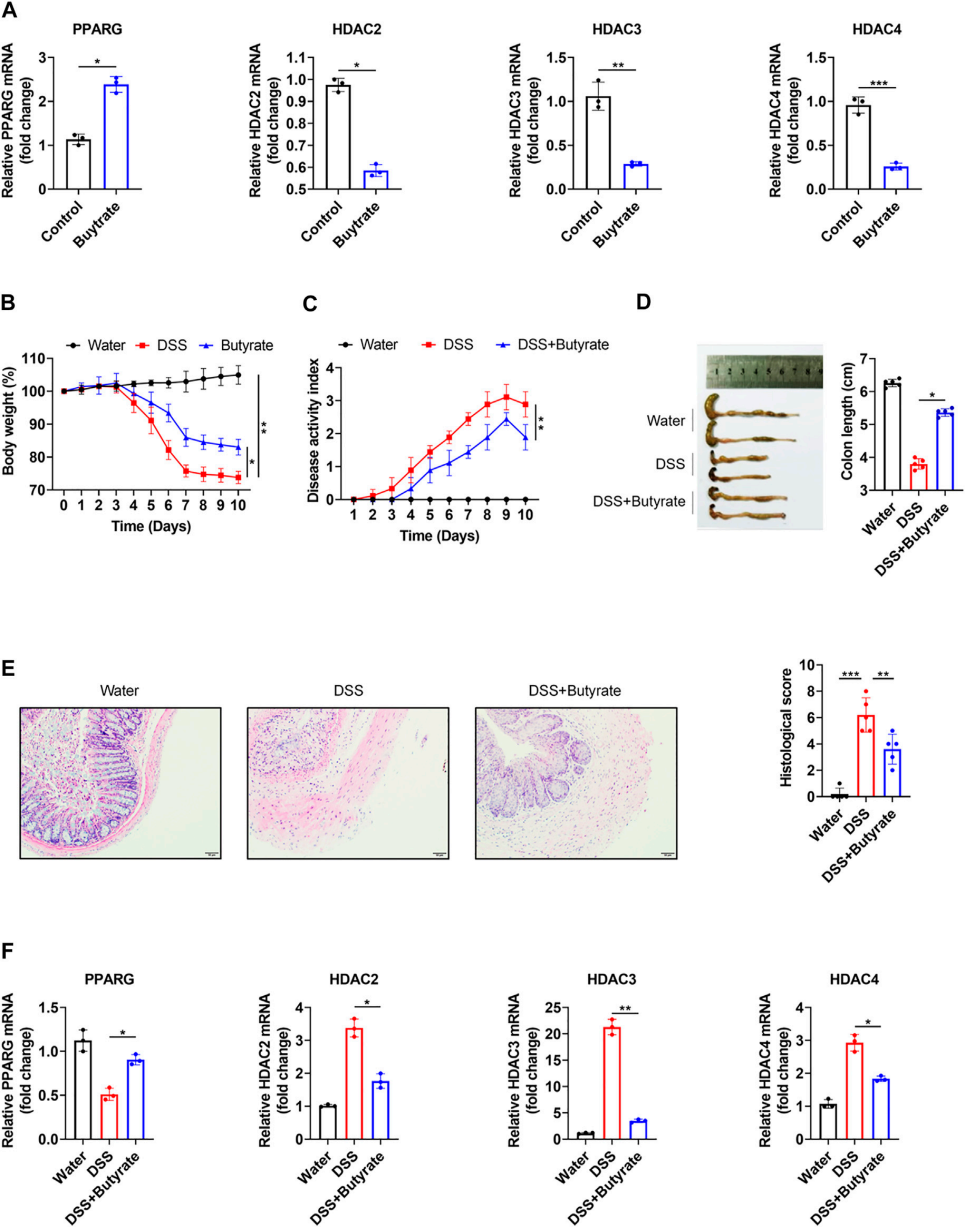
PPARG and HDACs can be regulated by butyrate. **(A)** PPARG, HDAC2, HDAC3 and HDAC4 mRNA levels were determined in Caco-2 cells treated with butyrate (5 mM) for 24 h; B57BL/6 mice were administered 2.5% DSS in drinking water for 7 days, followed by 3 days of water, in the presence or absence of butyrate (200 mM) (n = 5 per group). **(B)** Body weight, **(C)** DAI score, **(D)** colon length, **(E)** HE staining (200 × magnification) and histological scores or vechale. **(F)** PPARG, HDAC2, HDAC3, and HDAC4 mRNA levels in colonic epithelial cells. The data shown are the mean ± SD and represent three independent experiments, *p* values were calculated using unpaired t-test, *p < 0.05, **p < 0.01, ***p < 0.001, ns = not significant.

Our network of protein-metabolite analysis results indicated that PPARG and HDACs (HDAC2, HDAC3, HDAC4) can both interact with butyric acid (Figure 5A). Furthermore, molecular docking studies suggested that butyric acid can bind to PPARG and HDACs (Figures 5B,C). These data demonstrated that PPARG can interact with HDACs through butyric acid. Given the crucial role of metabolites in regulating protein function, we wondered whether butyric acid is upstream of PPARG and HDACs, which may contribute to the synergistic effects of the combination of 5-ASA and SAHA in the treatment of experimental colitis. Thus, we treated Caco-2 cells with butyrate (5 mM) and observed that the mRNA expression of PPARG was upregulated while the expression levels of HDAC2, HDAC3, and HDAC4 were downregulated (Figure 6A). In addition, body weight loss (Figure 6B), DAI score (Figure 6C), colonic shortening (Figure 6D) and colon pathology (Figure 6E) were improved in DSS-induced mice treated with butyrate (200 mM) compared with those receiving vehicle treatment. Additionally, DSS-induced colonic epithelial cells of mice treated with butyrate exhibited higher expression of PPARG and lower expression of HDAC2, HDAC3, and HDAC4 (Figure 6F). These results implied that the metabolite-butyric acid can target both PPARG and HDACs in experimental colitis. Thus, the synergistic effects of 5-ASA and SAHA in the treatment of experimental colitis may be associated with the metabolite- butyric acid.

## Discussion

In this study, we sought to identify synergistic drugs for 5-ASA to solve the problem of drug resistance and insufficient efficacy in certain cases. Through protein-metabolite interaction analysis, we identified potential drug targets. Then, we evaluated the possibility of synergistic effects on PPARG, a therapeutic target of 5-ASA, in the treatment of UC. Finally, we identified SAHA can exert synergistic effects with 5-ASA in the treatment of experimental colitis. One possible mechanism of these effects may be synergistic inhibition of the NF-kB signaling pathway. Moreover, the metabolite-butyric acid may participate in the synergistic effect. We validated their synergistic effects both *in vitro* and *in vivo* and the experimental results further demonstrated their synergistic effects.

At present, many in-silico methods have been developed to predict synergistic drug combinations especially in the field of cancer, such as breast cancer, lung cancer (Sun et al., 2015), and gastric cancer (Xiang et al., 2018). Researchers typically use currently known synergistic drug combinations as a seed dataset (Chen X. et al., 2016) and then develop different models or tools such as the Ranking-system of Anti-Cancer Synergy (RACS) (Sun et al., 2015), Network-based Laplacian regularized Least Square Synergistic (NLLSS) (Chen X. et al., 2016) and DrugComboRanker (Huang et al., 2014) to assess the synergistic potential of candidate drug pairs. Consequently, several synergistic drug combinations have been established. For example, the combined use of erlotinib and sorafenib has a major synergistic effect on the inhibition of MCF-7 breast cancer cell proliferation (Sun et al., 2015). However, these techniques may have limitations. 1) Some possible synergistic drugs may be missed due to limited drug pairs; 2) the molecular mechanism of synergistic effects of drug combinations is rarely disclosed; and 3) methods for the prediction of synergistic drug combinations of cancer diseases cannot be applied to other diseases. Therefore, we adopted protein-metabolite interactions to identify more potential drugs, starting with PPARG (Figure 1). Given that different drug targets involved in the same or associated pathways may produce synergistic effects (Jia et al., 2009), we further conducted KEGG and pathway-pathway interactions analysis to identify the targets that may participate in the same or a pathway related to PPARG. Then, we evaluated the possibility of synergy of these candidate drug targets and 5-ASA. Finally, we identified that HDAC2, HDAC3, and HDAC4 may exert excellent synergistic effects with PPARG (Figure 2). Moreover, PPARG was reported to participate in UC by inhibiting the NF-kB signaling pathway (Cao et al., 2018). Consequently, in our model, HDACs were also thought to regulate the NF-kB signaling pathway and synergize with PPARG. Recent studies have found that HDACs in colonic epithelial cells participate in the progression of colitis (Tsaprouni et al., 2011; Takeshima et al., 2012; Stylianou, 2013). Furthermore, HDAC inhibitors can exert anti-inflammatory effects through inhibiting the NF-kB and TGFβ1 signaling pathways (Chaturvedi et al., 2019; Friedrich et al., 2019).

According to the Therapeutic Target database, there are three approved drugs targeting histone deacetylase (HDAC), which include vorinostat (SAHA), sodium phenylbutyrate (PBA) and valproate (Wang et al., 2020). PBA is derived from the short-chain fatty acid-butyric acid and has been approved for the treatment of spinal muscular atrophy (Sun et al., 2019). Valproate was developed for the treatment of epilepsy (Bedlack et al., 2007). However, both PBA and valproate may cause significant damage to the liver and kidney (Perrine et al., 2007; Lee et al., 2015). Thus, we chose SAHA, an FDA-approved pan-HDACi that is clinically used to treat cutaneous T-cell lymphoma (Podar et al., 2009), as the ideal drug that may exert synergetic effects with 5-ASA. We validated their synergetic effects in UC through *in vivo* and *in vitro* experiments. The combination of 5-ASA and SAHA showed lower toxicity and mRNA expression of p65 in human colonic epithelial cell lines (Caco-2 and HCT-116) (Figure 3), and more efficiently alleviated symptoms of DSS-induced colitis than treatment with 5-ASA or SAHA alone (Figure 4). Thus, our approach suggests that SAHA combined with 5-ASA can produce synergistic effects in UC through synergistic inhibition of the NF-kB signaling pathway.

Our results demonstrated that butyric acid may bind to active sites of PPARG at His-323, Tyr-473, and His-449. Moreover, butyric acid can also interact with HDAC2 at Tyr-308, His-183, and His-146, interact with HDAC3 at Tyr-9 and interact with HDAC4 at His-842, His-802, and His-803. (Figure 5), Butyrate can upregulate the expression of PPARG while downregulating the expression of HDACs (Figure 6), which indicated that butyric acid may play an important role in the synergistic effects of 5-ASA and SAHA in the treatment of experimental colitis. Butyric acid is a short-chain fatty acid (SCFA) naturally produced by anaerobic bacteria (Ai et al., 2016). Recent studies have shown that SCFAs may participate in numerous biological processes (Ward et al., 2016). Moreover, a number of drugs have been reported to exert efficacy by influencing the production of SCFAs. For example, the natural plant extract-parthenolide (PTL) can exert a protective effect on DSS-induced colitis by regulating microbiota-derived SCFAs including acetate, propionate and butyrate (Liu et al., 2020). Thus, we wondered whether butyric acid is an intermediate link between 5-ASA targeting PPARG and SAHA targeting HDACs in the treatment of UC, which needs further study.

However, our strategy of identifying drugs that act synergistically with 5-ASA has the following limitations. First, protein-metabolite-protein analysis was conducted through the HMDB. HMDB Version 4.0 contains information on only 5,702 protein sequences and 114,186 associated metabolite entries (Wishart et al., 2018). However, millions of metabolite-protein interactions may exist (Piazza et al., 2018), which implies that not all target proteins can be associated with metabolite entries through the HMDB. Thus, more databases of protein-metabolite interactions should be considered in the future. Second, we focused more on therapeutic effects, but through our approach, side effects and toxicity of drug combinations couldn’t be determined. Therefore, the combination of SAHA and 5-ASA needs to be further validated *in vivo* and *in vitro* for toxicity and side effects. Third, through our strategy, we can identify only drug targets rather than direct drugs. Therefore, more combined drug-target networks or databases are needed to obtain the ideal candidate drugs.

In conclusion, our data suggested that the combination of SAHA and 5-ASA can exert significant synergistic effects in the treatment of UC, which may provide a solution for the problem of insufficient efficacy and drug resistance of 5-ASA in certain cases. One possible mechanism of the synergistic effects observed in this study may be their synergistic inhibition of the NF-kB signaling pathway and the metabolite- butyric acid may play a crucial role. In addition, our strategy can be applied to the identification of synergistic drug combinations for other diseases. However, whether the combination of SAHA and 5-ASA is suitable for clinical and the toxicity and side effects need to be further evaluated.

## Methods

### Protein-Metabolite Interaction Analysis and Network Construction

To analyze protein-metabolite interactions, we extracted information from the Human Metabolome Database (HMDB) on PPARG related metabolites and the proteins related to these metabolites. Using the TTD, we littered undruggable targets. Finally, Cystoscope software was used to visualize protein-metabolite interactions.

### KEGG Pathway Enrichment Analysis and Semantic Similarity of Gene Ontology Term Analysis

The “clusterProfiler”, “enrichplot”, and “ggplot2” packages were used to perform KEGG pathway enrichment analyses. Enrichment results with FDR (false discovery rate) < 0.05 were recognized as significant functional categories; The GOSemSim package (Yu et al., 2010) was used to determine semantic similarities of gene ontology terms.

### Gene-Overlapping Pathway-Pathway Interactions Analysis

Gene-overlapping pathway-pathway interactions (WWIs) were conducted as previously described (Chen D. et al., 2016). Briefly, when the number of shared genes between two pathways was significant against occasional conditions (by Fisher’s exact test, p-values of WWIs should be less than a certain threshold), they are regarded as one gene-overlapping WWI.

### Cell Counting Kit-8 (CCK-8) Analysis

The toxicity of 5-ASA and SAHA in Caco-2 or HCT-116 cells was determined by CCK8 assays. The Cell Counting Kit 8 (CCK8, BS350B, Biosharp, China) assay was performed following the manufacturer’s protocol. Briefly, at a density of 5,000 per well, Caco-2 or HCT-116 cells were plated onto 96-well plates and incubated overnight. On the second day, the cells were treated in combination or individually with 5-ASA and SAHA for 24 and 48 h. CCK-8 reagent (10 µl) was then added to each well and incubated for 2 h a 37°C in a 5% humidified CO_2_ atmosphere. Finally, the absorbance at 450 nm was measured using a microplate reader.

### Experimental Animals

C57BL/6 (wild type; WT) mice (male; 8–10 weeks of age; weighing 25–30 g) were purchased from Guangdong Medical Laboratory Animal Center. All mice were maintained under specific pathogen-free conditions (22 ± 2°C with 35–55% relative humidity on a 12-h light/dark cycle) with free access to food and water. All *in vivo* experiments were performed in accordance with protocols approved by the Institutional Animal Ethics Committee of the First Affiliated Hospital, Guangzhou University of Chinese Medicine (Ethics No. TCMF1-2018006).

### Induction of Experimental Colitis

Experimental colitis was induced by adding 2.5% DSS (molecular mass 36,000–50,000 Da; MP Biomedicals) to drinking water for 7 days, followed by regular drinking water for another 3 days.

### Butyrate Treatments

DSS-induced colitis mice were treated with butyrate (200 mM) in drinking water (Sun et al., 2018); HCT-116 and Caco-2 cells were stimulated with butyrate at a concentration of 5 mM (Olson et al., 2019).

### Isolation of Colonic Epithelial Cells (CECs)

Colonic epithelial cells (CECs) were isolated as previously described (Marchiori et al., 2019). Briefly, colon tissues were carefully washed with PBS and cut into 2–3 mm strips. The strips were then incubated for 10 min with 1 mM DTT at room temperature and for 20 min with 5 mM EDTA at 37°C. The CECs were obtained by centrifugation.

### Histological Score Analysis

Histological scores were analyzed as previously described (Zhou et al., 2019). Briefly, crypt architecture (normal, 0–severe crypt distortion with loss of entire crypts, 3), degree of inflammatory cell infiltration (normal, 0–dense inflammatory infiltrate, 3), muscle thickening (base of crypt sits on the muscularis mucosae, 0–marked muscle thickening, 3), crypt abscess (absent, 0–present, 1), and goblet cell depletion (absent, 0–present, 1) were recored. The total histological scores were derived by summing each individual score.

### Quantitative Real-Time Polymerase Chain Reaction

Trizol reagent was used to extract total RNA from Caco-2 and HCT-116 cells or colonic experimental colitis epithelial cells followed by cDNA synthesis. Using SYBR green gene expression assays on a Bio-Rad iCycler (Bio-Rad, Hercules, CA, United States ), quantitative PCRs were performed.

The sequences of the primers used were as follows:

**Table:** 

Gene name	Primer sequence
p65 (mice)	Forward: ACT​GCC​GGG​ATG​GCT​ACT​AT
	Reverse: TCT​GGA​TTC​GCT​GGC​TAA​TGG
IL-6 (mice)	Forward: CTG​CAA​GAG​ACT​TCC​ATC​CAG
	Reverse: AGT​GGT​ATA​GAC​AGG​TCT​GTT​GG
IL-1b (mice)	Forward: GAA​ATG​CCA​CCT​TTT​GAC​AGT​G
	Reverse: TGG​ATG​CTC​TCA​TCA​GGA​CAG
TNF-a (mice)	Forward: CTG​AAC​TTC​GGG​GTG​ATC​GG
	Reverse: GGC​TTG​TCA​CTC​GAA​TTT​TGA​GA
PPARG (human)	Forward: CGA​GAA​GGA​GAA​GCT​GTT​G
	Reverse: TCAGCGGGAAGGACTTTA
HDAC2 (human)	Forward: AGT​TGC​CCT​TGA​TTG​TGA​G
	Reverse: ATT​CTG​GAG​TGT​TCT​GGT​TTG
HDAC3 (human)	Forward: ATT​GGG​CTG​CTT​TAA​CCT​C
	Reverse: GGC​AAC​ATT​TCG​GAC​AGT​A
HDAC4 (human)	Forward: GGGAAAACGCAGCACAG
	Reverse: TCA​TCT​TTG​GCG​TCG​TAC​A
PPARG (mice)	Forward: CGA​GAA​GGA​GAA​GCT​GTT​G
	Reverse: TCAGCGGGAAGGACTTTA
HDAC2 (mice)	Forward: GGA​TGA​AGG​TGA​AGG​AGG​T
	Reverse: CAA​GGG​TTG​CTG​AGT​TGT​T
HDAC3 (mice)	Forward: GCGACCATGACAACGAC
	Reverse: AGA​CCT​GGG​GAA​ACC​ATA​C
HDAC4 (mice)	Forward: CCG​CTA​TGA​CGA​TGG​GAA​CT
	Reverse: CCA​CAT​CTG​GGG​CAA​ACT​C

### Statistical Analysis

The data were represented as the mean ± SD, and analysis was done using SPSS (version 20.0). To analyze significant differences between three or more classes, a one-way ANOVA test was used. ANOVA and multiple-ANOVA were used to analyze repeated measurement data. Statistical significance was set at *p* < 0.05.

## Data Availability

The raw data supporting the conclusions of this article will be made available by the authors, without undue reservation, to any qualified researcher.

## Abbreviations

5-ASA, 5-Aminosalicylic Acid; UC, Ulcerative Colitis; PPARG, Peroxisome Proliferator-Activated Receptor Gamma; KEGG, Kyoto Encyclopedia of Genes and Genome; SAHA, vorinostat; NF-kB, Nuclear factor kappaB; TNF, tumor necrosis factor; PPIs, Protein-Protein Interactions; TTD, Therapeutic Target database; HDAC, Histone Deacetylases.
