# Supplementary figures and images for: The Synergistic Effects of 5-Aminosalicylic Acid and Vorinostat in the Treatment of Ulcerative Colitis
Source: Front Pharmacol. 2021 May 21;12:625543. doi: 10.3389/fphar.2021.625543 (PMC8176098; doi:10.3389/fphar.2021.625543)

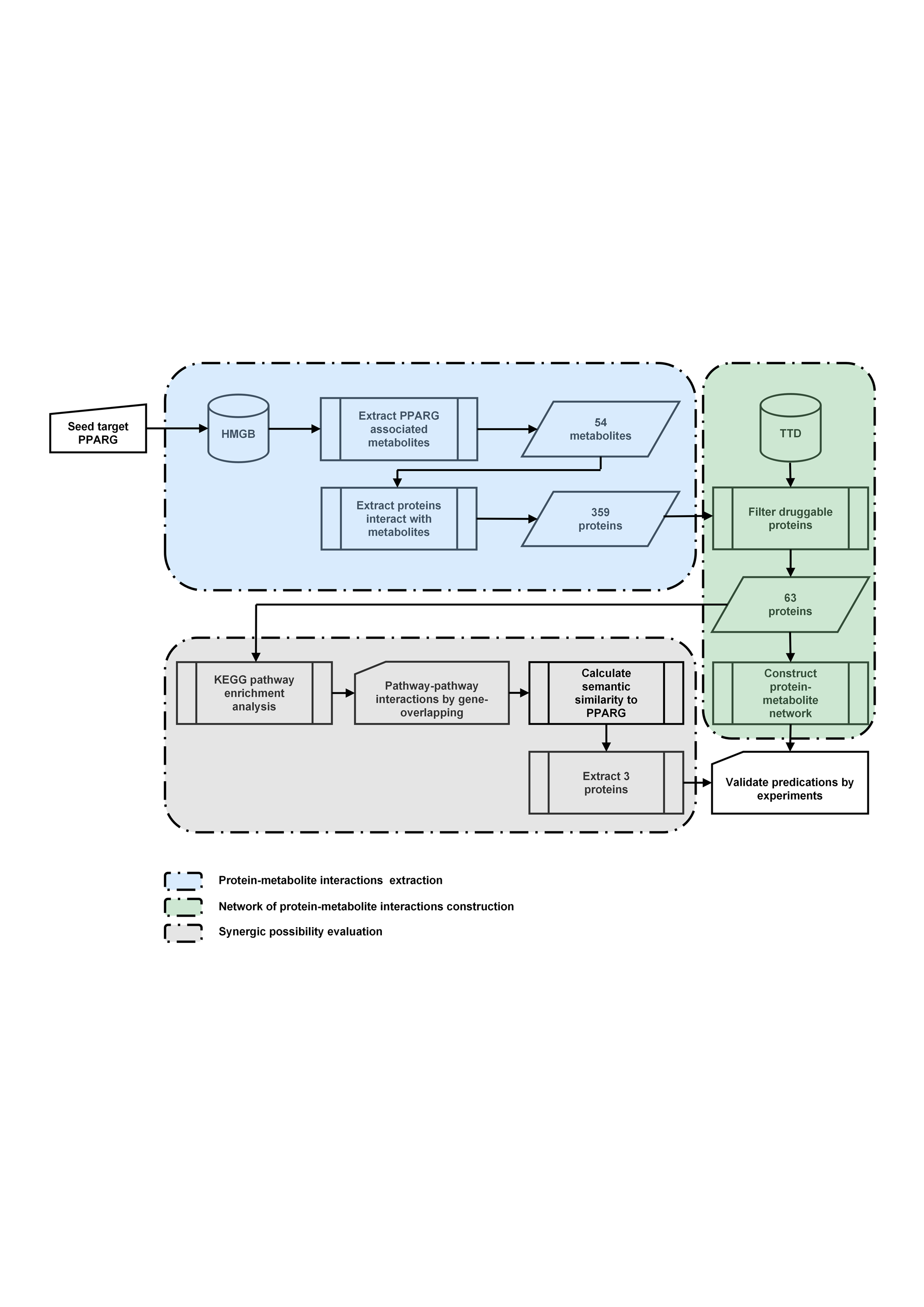

Supplement: Supplementary file 1 [file Image1.TIF]
