# Supplementary material for: The Synergistic Effects of 5-Aminosalicylic Acid and Vorinostat in the Treatment of Ulcerative Colitis
Source: Front Pharmacol. 2021 May 21;12:625543. doi: 10.3389/fphar.2021.625543 (PMC8176098; doi:10.3389/fphar.2021.625543)
Supplement: Supplementary file 2 [file DataSheet1.docx]

**Supplementary Materials**

**Figure S1. The overall workflow of this study.** The whole analysis process is made up of three parts: The protein-metabolite interactions extraction part (blue color) extracts formation about metabolites associated with PPARG and all proteins that associated with these metabolites through the Human Metabolome Database (HMDB); The network of protein-metabolite interactions construction part (grey color) screens out proteins which are druggable by using the Therapeutic Target Database (TTD) and constructs a network of protein-metabolite interactions; The synergic possibility evaluation part (grey color) conducts KEGG pathway enrichment and pathway-pathway interactions analysis on druggable protein targets. It also calculates semantic similarity scores of proteins compared with PPARG through the semantic GOSemSim package. Finally, 3 candidate proteins are screened out based on the literature and we validate our prediction results by experiments.

**Table S1. Network of metabolites and associated draggability proteins**

| **protein ID** | **gene** | **metabolites** |
| --- | --- | --- |
| P06276 | BCHE | Butyric acid |
| P01375 | TNF | Butyric acid |
| P37231 | PPARG | Butyric acid |
| P56524 | HDAC4 | Butyric acid |
| Q92769 | HDAC2 | Butyric acid |
| O15379 | HDAC3 | Butyric acid |
| P49327 | FASN | Palmitic acid |
| P47712 | PLA2G4A | Palmitic acid |
| P04054 | PLA2G1B | Palmitic acid |
| P14555 | PLA2G2A | Palmitic acid |
| P16233 | PNLIP | Palmitic acid |
| P07098 | LIPF | Palmitic acid |
| Q9Y5X9 | LIPG | Palmitic acid |
| P19835 | CEL | Palmitic acid |
| P06858 | LPL | Palmitic acid |
| P23141 | CES1 | Palmitic acid |
| P06276 | BCHE | Palmitic acid |
| Q05469 | LIPE | Palmitic acid |
| *P50897* | PPT1 | *Palmitic acid* |
| P37231 | PPARG | Palmitic acid |
| A2RRE8 | CPT1B | Palmitic acid |
| Q6L9M1 | PPARG | Palmitic acid |
| A5PLL0 | CPT1B | Palmitic acid |
| P49327 | FASN | Myristic acid |
| P47712 | PLA2G4A | Myristic acid |
| P04054 | PLA2G1B | Myristic acid |
| P14555 | PLA2G2A | Myristic acid |
| P16233 | PNLIP | Myristic acid |
| P07098 | LIPF | Myristic acid |
| Q9Y5X9 | LIPG | Myristic acid |
| P19835 | CEL | Myristic acid |
| P06858 | LPL | Myristic acid |
| P23141 | CES1 | Myristic acid |
| P06276 | BCHE | Myristic acid |
| Q05469 | LIPE | Myristic acid |
| P37231 | PPARG | Myristic acid |
| A2RRE8 | CPT1B | Myristic acid |
| Q6L9M1 | PPARG | Myristic acid |
| A5PLL0 | CPT1B | Myristic acid |
| P49327 | FASN | Pentadecanoic acid |
| P47712 | PLA2G4A | Pentadecanoic acid |
| P04054 | PLA2G1B | Pentadecanoic acid |
| P14555 | PLA2G2A | Pentadecanoic acid |
| P16233 | PNLIP | Pentadecanoic acid |
| P07098 | LIPF | Pentadecanoic acid |
| Q9Y5X9 | LIPG | Pentadecanoic acid |
| P19835 | CEL | Pentadecanoic acid |
| P06858 | LPL | Pentadecanoic acid |
| P23141 | CES1 | Pentadecanoic acid |
| P06276 | BCHE | Pentadecanoic acid |
| Q05469 | LIPE | Pentadecanoic acid |
| P37231 | PPARG | Pentadecanoic acid |
| A2RRE8 | CPT1B | Pentadecanoic acid |
| Q6L9M1 | PPARG | Pentadecanoic acid |
| A5PLL0 | CPT1B | Pentadecanoic acid |
| P49327 | FASN | Stearic acid |
| P47712 | PLA2G4A | Stearic acid |
| P04054 | PLA2G1B | Stearic acid |
| P14555 | PLA2G2A | Stearic acid |
| P16233 | PNLIP | Stearic acid |
| P07098 | LIPF | Stearic acid |
| Q9Y5X9 | LIPG | Stearic acid |
| P19835 | CEL | Stearic acid |
| P06858 | LPL | Stearic acid |
| P23141 | CES1 | Stearic acid |
| P06276 | BCHE | Stearic acid |
| Q05469 | LIPE | Stearic acid |
| P37231 | PPARG | Stearic acid |
| A2RRE8 | CPT1B | Stearic acid |
| Q6L9M1 | PPARG | Stearic acid |
| A5PLL0 | CPT1B | Stearic acid |
| P49327 | FASN | Tridecanoic acid |
| P47712 | PLA2G4A | Tridecanoic acid |
| P04054 | PLA2G1B | Tridecanoic acid |
| P14555 | PLA2G2A | Tridecanoic acid |
| P16233 | PNLIP | Tridecanoic acid |
| P07098 | LIPF | Tridecanoic acid |
| Q9Y5X9 | LIPG | Tridecanoic acid |
| P19835 | CEL | Tridecanoic acid |
| P06858 | LPL | Tridecanoic acid |
| P23141 | CES1 | Tridecanoic acid |
| P06276 | BCHE | Tridecanoic acid |
| Q05469 | LIPE | Tridecanoic acid |
| P37231 | PPARG | Tridecanoic acid |
| A2RRE8 | CPT1B | Tridecanoic acid |
| Q6L9M1 | PPARG | Tridecanoic acid |
| A5PLL0 | CPT1B | Tridecanoic acid |
| O75907 | DGAT1 | Methylbutyryl-CoA |
| O00767 | SCD | Methylbutyryl-CoA |
| O75908 | SOAT2 | Methylbutyryl-CoA |
| P35610 | SOAT1 | Methylbutyryl-CoA |
| P37231 | PPARG | Methylbutyryl-CoA |
| Q07869 | PPARA | Methylbutyryl-CoA |
| Q03181 | PPARD | Methylbutyryl-CoA |
| Q96PD7 | DGAT2 | Methylbutyryl-CoA |
| Q8NF37 | LPCAT1 | Methylbutyryl-CoA |
| P49327 | FASN | Arachidonic acid |
| P47712 | PLA2G4A | Arachidonic acid |
| P04054 | PLA2G1B | Arachidonic acid |
| P14555 | PLA2G2A | Arachidonic acid |
| P16233 | PNLIP | Arachidonic acid |
| P07098 | LIPF | Arachidonic acid |
| Q9Y5X9 | LIPG | Arachidonic acid |
| P19835 | CEL | Arachidonic acid |
| P06858 | LPL | Arachidonic acid |
| P23141 | CES1 | Arachidonic acid |
| P06276 | BCHE | Arachidonic acid |
| Q05469 | LIPE | Arachidonic acid |
| P09917 | ALOX5 | Arachidonic acid |
| P35354 | PTGS2 | Arachidonic acid |
| P23219 | PTGS1 | Arachidonic acid |
| P08684 | CYP3A4 | Arachidonic acid |
| P11712 | CYP2C9 | Arachidonic acid |
| Q16678 | CYP1B1 | Arachidonic acid |
| P11511 | CYP19A1 | Arachidonic acid |
| P11509 | CYP2A6 | Arachidonic acid |
| P37231 | PPARG | Arachidonic acid |
| Q6NWU0 | CYP2D6 | Arachidonic acid |
| A2RRE8 | CPT1B | Arachidonic acid |
| Q6L9M1 | PPARG | Arachidonic acid |
| A5PLL0 | CPT1B | Arachidonic acid |
| O75907 | DGAT1 | Octanoyl-CoA |
| O00767 | SCD | Octanoyl-CoA |
| O75908 | SOAT2 | Octanoyl-CoA |
| P35610 | SOAT1 | Octanoyl-CoA |
| P37231 | PPARG | Octanoyl-CoA |
| Q07869 | PPARA | Octanoyl-CoA |
| Q03181 | PPARD | Octanoyl-CoA |
| Q96PD7 | DGAT2 | Octanoyl-CoA |
| Q8NF37 | LPCAT1 | Octanoyl-CoA |
| P24752 | ACAT1 | Butyryl-CoA |
| O75907 | DGAT1 | Butyryl-CoA |
| O00767 | SCD | Butyryl-CoA |
| O75908 | SOAT2 | Butyryl-CoA |
| P35610 | SOAT1 | Butyryl-CoA |
| P37231 | PPARG | Butyryl-CoA |
| Q07869 | PPARA | Butyryl-CoA |
| Q03181 | PPARD | Butyryl-CoA |
| Q96PD7 | DGAT2 | Butyryl-CoA |
| Q8NF37 | LPCAT1 | Butyryl-CoA |
| O75907 | DGAT1 | Isovaleryl-CoA |
| O00767 | SCD | Isovaleryl-CoA |
| O75908 | SOAT2 | Isovaleryl-CoA |
| P35610 | SOAT1 | Isovaleryl-CoA |
| P37231 | PPARG | Isovaleryl-CoA |
| Q07869 | PPARA | Isovaleryl-CoA |
| Q03181 | PPARD | Isovaleryl-CoA |
| Q96PD7 | DGAT2 | Isovaleryl-CoA |
| Q8NF37 | LPCAT1 | Isovaleryl-CoA |
| O75907 | DGAT1 | Stearoyl-CoA |
| O00767 | SCD | Stearoyl-CoA |
| O75908 | SOAT2 | Stearoyl-CoA |
| P35610 | SOAT1 | Stearoyl-CoA |
| P37231 | PPARG | Stearoyl-CoA |
| Q07869 | PPARA | Stearoyl-CoA |
| Q03181 | PPARD | Stearoyl-CoA |
| Q96PD7 | DGAT2 | Stearoyl-CoA |
| Q8NF37 | LPCAT1 | Stearoyl-CoA |
| Q92793 | CREBBP | Acetyl-CoA |
| O00763 | ACACB | Acetyl-CoA |
| P49327 | FASN | Acetyl-CoA |
| P24752 | ACAT1 | Acetyl-CoA |
| O75907 | DGAT1 | Acetyl-CoA |
| O00767 | SCD | Acetyl-CoA |
| O75908 | SOAT2 | Acetyl-CoA |
| P35610 | SOAT1 | Acetyl-CoA |
| P37231 | PPARG | Acetyl-CoA |
| Q07869 | PPARA | Acetyl-CoA |
| Q03181 | PPARD | Acetyl-CoA |
| O00400 | SLC33A1 | Acetyl-CoA |
| Q96PD7 | DGAT2 | Acetyl-CoA |
| Q8NF37 | LPCAT1 | Acetyl-CoA |
| Q7L5N7 | LPCAT2 | Acetyl-CoA |
| O60502 | MGEA5 | Acetyl-CoA |
| O75907 | DGAT1 | Isobutyryl-CoA |
| O00767 | SCD | Isobutyryl-CoA |
| O75908 | SOAT2 | Isobutyryl-CoA |
| P35610 | SOAT1 | Isobutyryl-CoA |
| P37231 | PPARG | Isobutyryl-CoA |
| Q07869 | PPARA | Isobutyryl-CoA |
| Q03181 | PPARD | Isobutyryl-CoA |
| Q96PD7 | DGAT2 | Isobutyryl-CoA |
| Q8NF37 | LPCAT1 | Isobutyryl-CoA |
| O75907 | DGAT1 | Propionyl-CoA |
| O00767 | SCD | Propionyl-CoA |
| O75908 | SOAT2 | Propionyl-CoA |
| P35610 | SOAT1 | Propionyl-CoA |
| P37231 | PPARG | Propionyl-CoA |
| Q07869 | PPARA | Propionyl-CoA |
| Q03181 | PPARD | Propionyl-CoA |
| Q96PD7 | DGAT2 | Propionyl-CoA |
| Q8NF37 | LPCAT1 | Propionyl-CoA |
| O75907 | DGAT1 | Glutaconyl-CoA |
| O00767 | SCD | Glutaconyl-CoA |
| O75908 | SOAT2 | Glutaconyl-CoA |
| P35610 | SOAT1 | Glutaconyl-CoA |
| P37231 | PPARG | Glutaconyl-CoA |
| Q07869 | PPARA | Glutaconyl-CoA |
| Q03181 | PPARD | Glutaconyl-CoA |
| Q96PD7 | DGAT2 | Glutaconyl-CoA |
| Q8NF37 | LPCAT1 | Glutaconyl-CoA |
| O75907 | DGAT1 | Palmityl-CoA |
| *Q92523* | *CPT1B* | *Palmityl-CoA* |
| O00767 | SCD | Palmityl-CoA |
| O75908 | SOAT2 | Palmityl-CoA |
| P35610 | SOAT1 | Palmityl-CoA |
| *P50897* | *PPT1* | *Palmityl-CoA* |
| P37231 | PPARG | Palmityl-CoA |
| Q07869 | PPARA | Palmityl-CoA |
| Q03181 | PPARD | Palmityl-CoA |
| Q96PD7 | DGAT2 | Palmityl-CoA |
| Q8NF37 | LPCAT1 | Palmityl-CoA |
| P35354 | PTGS2 | Eicosapentaenoic acid |
| P23219 | PTGS1 | Eicosapentaenoic acid |
| O14842 | FFAR1 | Eicosapentaenoic acid |
| P37231 | PPARG | Eicosapentaenoic acid |
| Q8NER1 | TRPV1 | Eicosapentaenoic acid |
| Q03181 | PPARD | Eicosapentaenoic acid |
| O75907 | DGAT1 | Crotonoyl-CoA |
| O00767 | SCD | Crotonoyl-CoA |
| O75908 | SOAT2 | Crotonoyl-CoA |
| P35610 | SOAT1 | Crotonoyl-CoA |
| P37231 | PPARG | Crotonoyl-CoA |
| Q07869 | PPARA | Crotonoyl-CoA |
| Q03181 | PPARD | Crotonoyl-CoA |
| Q96PD7 | DGAT2 | Crotonoyl-CoA |
| Q8NF37 | LPCAT1 | Crotonoyl-CoA |
| O75907 | DGAT1 | Pristanoyl-CoA |
| *P05091* | *ALDH2* | *Pristanoyl-CoA* |
| O00767 | SCD | Pristanoyl-CoA |
| O75908 | SOAT2 | Pristanoyl-CoA |
| P35610 | SOAT1 | Pristanoyl-CoA |
| P37231 | PPARG | Pristanoyl-CoA |
| Q07869 | PPARA | Pristanoyl-CoA |
| Q03181 | PPARD | Pristanoyl-CoA |
| Q96PD7 | DGAT2 | Pristanoyl-CoA |
| Q8NF37 | LPCAT1 | Pristanoyl-CoA |
| P49327 | FASN | Arachidic acid |
| P47712 | PLA2G4A | Arachidic acid |
| P04054 | PLA2G1B | Arachidic acid |
| P14555 | PLA2G2A | Arachidic acid |
| P16233 | PNLIP | Arachidic acid |
| P07098 | LIPF | Arachidic acid |
| Q9Y5X9 | LIPG | Arachidic acid |
| P19835 | CEL | Arachidic acid |
| P06858 | LPL | Arachidic acid |
| P23141 | CES1 | Arachidic acid |
| P06276 | BCHE | Arachidic acid |
| Q05469 | LIPE | Arachidic acid |
| P37231 | PPARG | Arachidic acid |
| A2RRE8 | CPT1B | Arachidic acid |
| Q6L9M1 | PPARG | Arachidic acid |
| A5PLL0 | CPT1B | Arachidic acid |
| P49327 | FASN | Heptadecanoic acid |
| P47712 | PLA2G4A | Heptadecanoic acid |
| P04054 | PLA2G1B | Heptadecanoic acid |
| P14555 | PLA2G2A | Heptadecanoic acid |
| P16233 | PNLIP | Heptadecanoic acid |
| P07098 | LIPF | Heptadecanoic acid |
| Q9Y5X9 | LIPG | Heptadecanoic acid |
| P19835 | CEL | Heptadecanoic acid |
| P06858 | LPL | Heptadecanoic acid |
| P23141 | CES1 | Heptadecanoic acid |
| P06276 | BCHE | Heptadecanoic acid |
| Q05469 | LIPE | Heptadecanoic acid |
| P37231 | PPARG | Heptadecanoic acid |
| A2RRE8 | CPT1B | Heptadecanoic acid |
| Q6L9M1 | PPARG | Heptadecanoic acid |
| A5PLL0 | CPT1B | Heptadecanoic acid |
| O75907 | DGAT1 | Acrylyl-CoA |
| O00767 | SCD | Acrylyl-CoA |
| O75908 | SOAT2 | Acrylyl-CoA |
| P35610 | SOAT1 | Acrylyl-CoA |
| P37231 | PPARG | Acrylyl-CoA |
| Q07869 | PPARA | Acrylyl-CoA |
| Q03181 | PPARD | Acrylyl-CoA |
| Q96PD7 | DGAT2 | Acrylyl-CoA |
| Q8NF37 | LPCAT1 | Acrylyl-CoA |
| O75907 | DGAT1 | Hexanoyl-CoA |
| O00767 | SCD | Hexanoyl-CoA |
| O75908 | SOAT2 | Hexanoyl-CoA |
| P35610 | SOAT1 | Hexanoyl-CoA |
| P37231 | PPARG | Hexanoyl-CoA |
| Q07869 | PPARA | Hexanoyl-CoA |
| Q03181 | PPARD | Hexanoyl-CoA |
| Q96PD7 | DGAT2 | Hexanoyl-CoA |
| Q8NF37 | LPCAT1 | Hexanoyl-CoA |
| O75907 | DGAT1 | Lauroyl-CoA |
| O00767 | SCD | Lauroyl-CoA |
| O75908 | SOAT2 | Lauroyl-CoA |
| P35610 | SOAT1 | Lauroyl-CoA |
| P37231 | PPARG | Lauroyl-CoA |
| Q07869 | PPARA | Lauroyl-CoA |
| Q03181 | PPARD | Lauroyl-CoA |
| Q96PD7 | DGAT2 | Lauroyl-CoA |
| Q8NF37 | LPCAT1 | Lauroyl-CoA |
| O75907 | DGAT1 | (2E)-Dodecenoyl-CoA |
| O00767 | SCD | (2E)-Dodecenoyl-CoA |
| O75908 | SOAT2 | (2E)-Dodecenoyl-CoA |
| P35610 | SOAT1 | (2E)-Dodecenoyl-CoA |
| P37231 | PPARG | (2E)-Dodecenoyl-CoA |
| Q07869 | PPARA | (2E)-Dodecenoyl-CoA |
| Q03181 | PPARD | (2E)-Dodecenoyl-CoA |
| Q96PD7 | DGAT2 | (2E)-Dodecenoyl-CoA |
| Q8NF37 | LPCAT1 | (2E)-Dodecenoyl-CoA |
| O75907 | DGAT1 | trans-2-Hexenoyl-CoA |
| O00767 | SCD | trans-2-Hexenoyl-CoA |
| O75908 | SOAT2 | trans-2-Hexenoyl-CoA |
| P35610 | SOAT1 | trans-2-Hexenoyl-CoA |
| P37231 | PPARG | trans-2-Hexenoyl-CoA |
| Q07869 | PPARA | trans-2-Hexenoyl-CoA |
| Q03181 | PPARD | trans-2-Hexenoyl-CoA |
| Q96PD7 | DGAT2 | trans-2-Hexenoyl-CoA |
| Q8NF37 | LPCAT1 | trans-2-Hexenoyl-CoA |
| O75907 | DGAT1 | (2E)-Hexadecenoyl-CoA |
| O00767 | SCD | (2E)-Hexadecenoyl-CoA |
| O75908 | SOAT2 | (2E)-Hexadecenoyl-CoA |
| P35610 | SOAT1 | (2E)-Hexadecenoyl-CoA |
| P37231 | PPARG | (2E)-Hexadecenoyl-CoA |
| Q07869 | PPARA | (2E)-Hexadecenoyl-CoA |
| Q03181 | PPARD | (2E)-Hexadecenoyl-CoA |
| Q96PD7 | DGAT2 | (2E)-Hexadecenoyl-CoA |
| Q8NF37 | LPCAT1 | (2E)-Hexadecenoyl-CoA |
| O75907 | DGAT1 | (2E)-Tetradecenoyl-CoA |
| O00767 | SCD | (2E)-Tetradecenoyl-CoA |
| O75908 | SOAT2 | (2E)-Tetradecenoyl-CoA |
| P35610 | SOAT1 | (2E)-Tetradecenoyl-CoA |
| P37231 | PPARG | (2E)-Tetradecenoyl-CoA |
| Q07869 | PPARA | (2E)-Tetradecenoyl-CoA |
| Q03181 | PPARD | (2E)-Tetradecenoyl-CoA |
| Q96PD7 | DGAT2 | (2E)-Tetradecenoyl-CoA |
| Q8NF37 | LPCAT1 | (2E)-Tetradecenoyl-CoA |
| O75907 | DGAT1 | (2E)-Decenoyl-CoA |
| O00767 | SCD | (2E)-Decenoyl-CoA |
| O75908 | SOAT2 | (2E)-Decenoyl-CoA |
| P35610 | SOAT1 | (2E)-Decenoyl-CoA |
| P37231 | PPARG | (2E)-Decenoyl-CoA |
| Q07869 | PPARA | (2E)-Decenoyl-CoA |
| Q03181 | PPARD | (2E)-Decenoyl-CoA |
| Q96PD7 | DGAT2 | (2E)-Decenoyl-CoA |
| Q8NF37 | LPCAT1 | (2E)-Decenoyl-CoA |
| O75907 | DGAT1 | (2E)-Octenoyl-CoA |
| O00767 | SCD | (2E)-Octenoyl-CoA |
| O75908 | SOAT2 | (2E)-Octenoyl-CoA |
| P35610 | SOAT1 | (2E)-Octenoyl-CoA |
| P37231 | PPARG | (2E)-Octenoyl-CoA |
| Q07869 | PPARA | (2E)-Octenoyl-CoA |
| Q03181 | PPARD | (2E)-Octenoyl-CoA |
| Q96PD7 | DGAT2 | (2E)-Octenoyl-CoA |
| Q8NF37 | LPCAT1 | (2E)-Octenoyl-CoA |
| O75907 | DGAT1 | 3Z-dodecenoyl-CoA |
| O00767 | SCD | 3Z-dodecenoyl-CoA |
| O75908 | SOAT2 | 3Z-dodecenoyl-CoA |
| P35610 | SOAT1 | 3Z-dodecenoyl-CoA |
| P37231 | PPARG | 3Z-dodecenoyl-CoA |
| Q07869 | PPARA | 3Z-dodecenoyl-CoA |
| Q03181 | PPARD | 3Z-dodecenoyl-CoA |
| Q96PD7 | DGAT2 | 3Z-dodecenoyl-CoA |
| Q8NF37 | LPCAT1 | 3Z-dodecenoyl-CoA |
| O75907 | DGAT1 | Eicosanoyl-CoA |
| O00767 | SCD | Eicosanoyl-CoA |
| O75908 | SOAT2 | Eicosanoyl-CoA |
| P35610 | SOAT1 | Eicosanoyl-CoA |
| P37231 | PPARG | Eicosanoyl-CoA |
| Q07869 | PPARA | Eicosanoyl-CoA |
| Q03181 | PPARD | Eicosanoyl-CoA |
| Q96PD7 | DGAT2 | Eicosanoyl-CoA |
| Q8NF37 | LPCAT1 | Eicosanoyl-CoA |
| P04035 | HMGCR | Atorvastatin |
| P08684 | CYP3A4 | Atorvastatin |
| P05121 | SERPINE1 | Atorvastatin |
| P13500 | CCL2 | Atorvastatin |
| P01375 | TNF | Atorvastatin |
| P02741 | CRP | Atorvastatin |
| P04114 | APOB | Atorvastatin |
| P15692 | VEGFA | Atorvastatin |
| P29965 | CD40LG | Atorvastatin |
| P37231 | PPARG | Atorvastatin |
| Q07869 | PPARA | Atorvastatin |
| P11712 | CYP2C9 | Rosiglitazone |
| P37231 | PPARG | Rosiglitazone |
| O75907 | DGAT1 | Decanoyl-CoA (n-C10:0CoA) |
| O00767 | SCD | Decanoyl-CoA (n-C10:0CoA) |
| O75908 | SOAT2 | Decanoyl-CoA (n-C10:0CoA) |
| P35610 | SOAT1 | Decanoyl-CoA (n-C10:0CoA) |
| P37231 | PPARG | Decanoyl-CoA (n-C10:0CoA) |
| Q07869 | PPARA | Decanoyl-CoA (n-C10:0CoA) |
| Q03181 | PPARD | Decanoyl-CoA (n-C10:0CoA) |
| Q96PD7 | DGAT2 | Decanoyl-CoA (n-C10:0CoA) |
| Q8NF37 | LPCAT1 | Decanoyl-CoA (n-C10:0CoA) |
| P49327 | FASN | Heptadecanoyl CoA |
| P47712 | PLA2G4A | Heptadecanoyl CoA |
| P04054 | PLA2G1B | Heptadecanoyl CoA |
| P14555 | PLA2G2A | Heptadecanoyl CoA |
| P16233 | PNLIP | Heptadecanoyl CoA |
| P07098 | LIPF | Heptadecanoyl CoA |
| Q9Y5X9 | LIPG | Heptadecanoyl CoA |
| P19835 | CEL | Heptadecanoyl CoA |
| O75907 | DGAT1 | Heptadecanoyl CoA |
| P06858 | LPL | Heptadecanoyl CoA |
| O00767 | SCD | Heptadecanoyl CoA |
| P23141 | CES1 | Heptadecanoyl CoA |
| O75908 | SOAT2 | Heptadecanoyl CoA |
| P35610 | SOAT1 | Heptadecanoyl CoA |
| P06276 | BCHE | Heptadecanoyl CoA |
| Q05469 | LIPE | Heptadecanoyl CoA |
| P37231 | PPARG | Heptadecanoyl CoA |
| Q07869 | PPARA | Heptadecanoyl CoA |
| Q03181 | PPARD | Heptadecanoyl CoA |
| Q96PD7 | DGAT2 | Heptadecanoyl CoA |
| Q8NF37 | LPCAT1 | Heptadecanoyl CoA |
| A2RRE8 | CPT1B | Heptadecanoyl CoA |
| Q6L9M1 | PPARG | Heptadecanoyl CoA |
| A5PLL0 | CPT1B | Heptadecanoyl CoA |
| O75907 | DGAT1 | Tetracosanoyl-CoA |
| O00767 | SCD | Tetracosanoyl-CoA |
| O75908 | SOAT2 | Tetracosanoyl-CoA |
| P35610 | SOAT1 | Tetracosanoyl-CoA |
| P37231 | PPARG | Tetracosanoyl-CoA |
| Q07869 | PPARA | Tetracosanoyl-CoA |
| Q03181 | PPARD | Tetracosanoyl-CoA |
| Q96PD7 | DGAT2 | Tetracosanoyl-CoA |
| Q8NF37 | LPCAT1 | Tetracosanoyl-CoA |
| O75907 | DGAT1 | trans-Octadec-2-enoyl-CoA |
| O00767 | SCD | trans-Octadec-2-enoyl-CoA |
| O75908 | SOAT2 | trans-Octadec-2-enoyl-CoA |
| P35610 | SOAT1 | trans-Octadec-2-enoyl-CoA |
| P37231 | PPARG | trans-Octadec-2-enoyl-CoA |
| Q07869 | PPARA | trans-Octadec-2-enoyl-CoA |
| Q03181 | PPARD | trans-Octadec-2-enoyl-CoA |
| Q96PD7 | DGAT2 | trans-Octadec-2-enoyl-CoA |
| Q8NF37 | LPCAT1 | trans-Octadec-2-enoyl-CoA |
| O75907 | DGAT1 | Methylhexanoyl-CoA |
| O00767 | SCD | Methylhexanoyl-CoA |
| O75908 | SOAT2 | Methylhexanoyl-CoA |
| P35610 | SOAT1 | Methylhexanoyl-CoA |
| P37231 | PPARG | Methylhexanoyl-CoA |
| Q07869 | PPARA | Methylhexanoyl-CoA |
| Q03181 | PPARD | Methylhexanoyl-CoA |
| Q96PD7 | DGAT2 | Methylhexanoyl-CoA |
| Q8NF37 | LPCAT1 | Methylhexanoyl-CoA |
| O75907 | DGAT1 | Heptanoyl-CoA |
| O00767 | SCD | Heptanoyl-CoA |
| O75908 | SOAT2 | Heptanoyl-CoA |
| P35610 | SOAT1 | Heptanoyl-CoA |
| P37231 | PPARG | Heptanoyl-CoA |
| Q07869 | PPARA | Heptanoyl-CoA |
| Q03181 | PPARD | Heptanoyl-CoA |
| Q96PD7 | DGAT2 | Heptanoyl-CoA |
| Q8NF37 | LPCAT1 | Heptanoyl-CoA |
| O75907 | DGAT1 | Nonanoyl-CoA |
| O00767 | SCD | Nonanoyl-CoA |
| O75908 | SOAT2 | Nonanoyl-CoA |
| P35610 | SOAT1 | Nonanoyl-CoA |
| P37231 | PPARG | Nonanoyl-CoA |
| Q07869 | PPARA | Nonanoyl-CoA |
| Q03181 | PPARD | Nonanoyl-CoA |
| Q96PD7 | DGAT2 | Nonanoyl-CoA |
| Q8NF37 | LPCAT1 | Nonanoyl-CoA |
| O75907 | DGAT1 | Pentanoyl-CoA |
| O00767 | SCD | Pentanoyl-CoA |
| O75908 | SOAT2 | Pentanoyl-CoA |
| P35610 | SOAT1 | Pentanoyl-CoA |
| P37231 | PPARG | Pentanoyl-CoA |
| Q07869 | PPARA | Pentanoyl-CoA |
| Q03181 | PPARD | Pentanoyl-CoA |
| Q96PD7 | DGAT2 | Pentanoyl-CoA |
| Q8NF37 | LPCAT1 | Pentanoyl-CoA |
| O75907 | DGAT1 | Undecanoyl-CoA |
| O00767 | SCD | Undecanoyl-CoA |
| O75908 | SOAT2 | Undecanoyl-CoA |
| P35610 | SOAT1 | Undecanoyl-CoA |
| P37231 | PPARG | Undecanoyl-CoA |
| Q07869 | PPARA | Undecanoyl-CoA |
| Q03181 | PPARD | Undecanoyl-CoA |
| Q96PD7 | DGAT2 | Undecanoyl-CoA |
| Q8NF37 | LPCAT1 | Undecanoyl-CoA |
| P05164 | MPO | Mesalazine |
| P09917 | ALOX5 | Mesalazine |
| P35354 | PTGS2 | Mesalazine |
| P23219 | PTGS1 | Mesalazine |
| O14920 | IKBKB | Mesalazine |
| P37231 | PPARG | Mesalazine |
| P23219 | PTGS1 | Nateglinide |
| P08684 | CYP3A4 | Nateglinide |
| P11712 | CYP2C9 | Nateglinide |
| P10635 | CYP2D6 | Nateglinide |
| P37231 | PPARG | Nateglinide |
| Q09428 | ABCC8 | Nateglinide |
| P24752 | ACAT1 | Sulfasalazine |
| P09917 | ALOX5 | Sulfasalazine |
| P35354 | PTGS2 | Sulfasalazine |
| P23219 | PTGS1 | Sulfasalazine |
| O14920 | IKBKB | Sulfasalazine |
| P37231 | PPARG | Sulfasalazine |
| Q9UNQ0 | ABCG2 | Sulfasalazine |
| Q96NT5 | SLC46A1 | Sulfasalazine |
| P08684 | CYP3A4 | Repaglinide |
| P37231 | PPARG | Repaglinide |
| Q09428 | ABCC8 | Repaglinide |
| P08183 | ABCB1 | Telmisartan |
| P30556 | AGTR1 | Telmisartan |
| P37231 | PPARG | Telmisartan |
| Q9UNQ0 | ABCG2 | Telmisartan |
| P09917 | ALOX5 | Balsalazide |
| P35354 | PTGS2 | Balsalazide |
| P23219 | PTGS1 | Balsalazide |
| P37231 | PPARG | Balsalazide |
| P08684 | CYP3A4 | Glipizide |
| P11712 | CYP2C9 | Glipizide |
| P37231 | PPARG | Glipizide |
| Q09428 | ABCC8 | Glipizide |
| P23219 | PTGS1 | Pioglitazone |
| P08684 | CYP3A4 | Pioglitazone |
| P11712 | CYP2C9 | Pioglitazone |
| P10635 | CYP2D6 | Pioglitazone |
| P37231 | PPARG | Pioglitazone |
| P37231 | PPARG | Mitiglinide |
| Q09428 | ABCC8 | Mitiglinide |
| P08684 | CYP3A4 | Bezafibrate |
| P37231 | PPARG | Bezafibrate |
| Q07869 | PPARA | Bezafibrate |
| Q03181 | PPARD | Bezafibrate |

**Table S2. The full semantic similarity ranking list based on molecular function (MF) and component (CC)**

| **Protein** | **semantic similarity** |
| --- | --- |
| PPARG | 1 |
| PPARA | 0.84510946 |
| PPARD | 0.838858749 |
| CREBBP | 0.727696365 |
| HDAC2 | 0.725939391 |
| HDAC4 | 0.714321356 |
| HDAC3 | 0.660686007 |
| ACACB | 0.614413542 |
| PTGS2 | 0.586432434 |
| IKBKB | 0.578058821 |
| ALOX5 | 0.575681335 |
| CYP2A6 | 0.55211593 |
| FASN | 0.544189305 |
| CYP2D6 | 0.54201476 |
| CYP3A4 | 0.536128716 |
| CYP2C9 | 0.530423416 |
| SCD | 0.529021739 |
| PPT1 | 0.528124038 |
| APOB | 0.527485545 |
| VEGFA | 0.523812944 |
| ABCG2 | 0.521888877 |
| LIPE | 0.511163379 |
| LIPF | 0.510529137 |
| HMGCR | 0.510118614 |
| TRPV1 | 0.509881359 |
| TNF | 0.509487978 |
| CPT1B | 0.509099204 |
| SOAT1 | 0.502808114 |
| MPO | 0.498623104 |
| ACAT1 | 0.494243867 |
| CEL | 0.493315315 |
| PLA2G1B | 0.489428238 |
| BCHE | 0.483006211 |
| CYP19A1 | 0.47520101 |
| CYP1B1 | 0.470824808 |
| PTGS1 | 0.452910587 |
| DGAT2 | 0.431149626 |
| LPL | 0.430546165 |
| DGAT1 | 0.427286789 |
| SERPINE1 | 0.417118688 |
| PLA2G4A | 0.416075714 |
| CCL2 | 0.41338118 |
| PLA2G2A | 0.396056814 |
| AGTR1 | 0.39581814 |
| CD40LG | 0.391918359 |
| ABCB1 | 0.382115166 |
| ABCC8 | 0.362129811 |
| FFAR1 | 0.354970421 |
| SOAT2 | 0.352664146 |
| LPCAT2 | 0.348633045 |
| CRP | 0.346684294 |
| LPCAT1 | 0.345320141 |
| ALDH2 | 0.342994169 |
| LIPG | 0.341381019 |
| SLC46A1 | 0.308090896 |
| CES1 | 0.286966897 |
| PNLIP | 0.24827807 |
| SLC33A1 | 0.233456634 |

**Table S3. Databases used and their details**

| **Name** | **Version** | **Access date** |
| --- | --- | --- |
| Human Metabolome Database | 4.0 | 2020-07 |
| Therapeutic Target Database | June 1^st^ ,2020 | 2020-08 |
